# Naomi Cordero Broering (1929-2023)

**DOI:** 10.5195/jmla.2024.1705

**Published:** 2024-01-16

**Authors:** Alan Carr, Michelle Kraft

**Affiliations:** 1 alanfcarr@hotmail.com, Former Associate Director, NNLM Pacific Southwest Region, UCLA Louise Darling Biomedical Library, Los Angeles, CA; 2 kraftm@ccf.org, Co-Lead Editor, Journal of the Medical Library Association, Medical Library Director, Cleveland Clinic Health System, Floyd D. Loop Alumni Library, Cleveland, OH

After a long and illustrious career in librarianship, Naomi Cordero Broering, MA, MLS, AHIP, FMLA, died on January 11, 2023. Born in New York City to Puerto Rican parents, Naomi served as MLA's first Hispanic/Latinx president in 1996/97, and in 2003, she received MLA's Marcia C. Noyes Award, MLA's highest professional distinction.

The arc of Naomi's career spanned several years, culminating as dean of libraries at Pacific College of Oriental Medicine (PCOM) in San Diego, where she retired in 2018. While at PCOM, she received numerous outreach funding awards from the National Network Libraries of Medicine (NNLM), Pacific Southwest Region (PSR). According to Alan Carr, retired, associate director, NNLM, PSR, Naomi's outreach was highly impactful and encompassed diverse locations and populations including Hispanics, African Americans, Native Americans, Pacific Islanders, and individuals living in areas with a high incidence of HIV. Her fluency in Spanish enhanced her outreach efforts.

Naomi's husband, Lieutenant Commander Gregory Chauncey, U.S. Navy, retired, often joined her in the exhibit hall at MLA meetings to discuss outreach projects. In 2015, they established and endowed the MLA Naomi C. Broering Hispanic Heritage Grant (now Latinx Heritage Grant) with Naomi being the first recipient. The grant commemorates Naomi's more than four decades of contributions to the profession and is awarded annually to a person of Latinx ethnicity or a person who has an interest in Latinx community information services.

Prior to Naomi's tenure at PCOM, she was the executive director at the Houston Academy of Medicine-Texas Medical Center (HAM/TMC) Library and director, NNLM South Central Region, from 1996-1999. Serving 10 medical schools and an extensive network of hospitals at the time, HAM/TMC Library ranked close to the top due to the size of their budget, personnel, and building, as well as the number of print and electronic resources.

Before coming to HAM/TMC, Naomi left an indelible mark while serving as director, Biomedical Information Resources Center and medical center librarian, Georgetown University Medical Center, Dahlgren Memorial Library from 1975-1996. Under her leadership, the Georgetown University Library Information System (LIS) [1] was among the first integrated online systems developed to automate typical library functions such as acquisitions, catalog, serials, circulation, and bibliographic management (including the mini–MEDLINE System) [2]. LIS and mini-MEDLINE were marketed and sold to libraries across the country.

In addition to serving as MLA President and member of the Board of Directors, Naomi was a Distinguished Member of the Academy of Health Information Professionals, a Fellow of MLA and the American College of Medical Informatics, and editor of the Bulletin of the Medical Library Association (BMLA). Naomi was a founding member and served as secretary of the Friends of the National Library of Medicine. She was also active in several other national associations including the American Society for Information Sciences, American Library Association, American Medical Informatics Association, and the Special Library Association (SLA). She received numerous honors including the SLA Winifred Sewell Award, SLA Professional Award, and the MLA Frank B. Rogers Information Advancement Award.

Leading up to her presidential address in 1996, Naomi presided at all the events of the annual meeting due to the absence of Jana Bradley, who was president at that time. Naomi's presidential address began with a challenge, “Today, I want to ask you to think about what you will be doing in the twenty-first century - in the year 2000 and beyond.” She challenged the audience to say “I will serve a larger user base … I will be transmitting health care information to every member of the family … much of my work will be outside the library … it will be a virtual library …” Naomi's words from 1996 resonate with contemporary themes such as health informatics, the NNLM All of Us Program Center, library liaisons, participation in campus/hospital committees, and the development of virtual libraries for native populations.

In the July 1996 BMLA presidential profile of Naomi, Susan Crawford stated, “She was one of the pioneers in implementing the integrated academic (now advanced) information management system (IAMS). Other technical accomplishments include an electronic textbook in human physiology and BIOSYNTHESIS that integrates multiple databases for access through a single gateway. The MAClinical Workstation is a project to develop computer workstations for medical students to prepare them to use computers in their future medical practice.”

Naomi was a prolific author, successful grant writer, and ardent supporter of the NLM. She authored over 200 articles and was awarded numerous grants and contracts from the NLM across three NNLM regions. According to the NIH Reporter, Naomi received $5,690,638 in funding from 1985-1995 which was significant for Dahlgren Memorial Library. She leaves a legacy within the academy as a collaborator, visionary, advocate, trailblazer, prolific writer, successful grantee, and supporter of the goals and aspirations of MLA and the NLM. In the July 1992 editorial in BMLA, Susan Crawford introduced Naomi as the new editor with these words, “Vision, creativity, and a great sense of timing.”
